# Woodward on Cancer

**Published:** 1874-02

**Authors:** 


### Bibliographical.

Toner Lecture No. 1.—On the Structure of Cancerous Tumors, and the mode in which adjacent parts are invaded. By J. J. Woodward, Assistant Surgeon United States Army. Delivered March 28, 1873. Washington: Smithsonian Institute. November, 1873. Octavo, pp. 40.

In the limited time and space at our disposal we cannot undertake a systematic analysis of this able lecture of Dr. Woodward, as we had hoped to do, for the benefit of our readers. We can only append the practical conclusions deduced from his investigations, and given in his own words, advising the interested reader to apply for the pamphlet at the Smithsonian Institute.

"If now I recur, as I promised to do, to the question of diagnosis, I think you will understand, without any detailed repetition of the descriptions I have given, that in the present state of our knowledge we ought, on anatomical grounds, to call any growth a cancer, at the margins of which we find the nucleated or cell cylinders I have described, associated with the infiltration of small cells in the intervening connective tissue. It is not necessary to wait till the cells of the cylinders have acquired any great size in order to arrive at an opinion.

"And at this point the important question of prognosis thrusts itself again upon our attention. Can we be sure that a growth which has the anatomy of cancer will have the history that is usually indicated by the word malignant; that it will ulcerate if left to itself; that it will recur if extirpated; that in either case similar growths will develop in some of the internal organs; and that the patient will surely die from this cause, if not from the primary disease? On the other hand, if a tumor be extirpated, which does not possess the anatomy of cancer, in which no proper cancer cylinders have been found—as, for example, in the first case of mammary tumor to which I alluded this evening—can we be sure that the growth would not have ultimately acquired the cancerous anatomy if it had been let alone? Can we be sure that it will not, in spite of its timely extirpation, recur and prove fatal?

"A proper discussion of these important questions, based upon a consideration of all the evidence, would require much time and thought, and be a work of no small labor and difficulty. Of course it cannot be undertaken in the present lecture. Nevertheless, it may not be amiss to state that the general tenor of surgical experience would seem to give a negative answer to both these important questions. With regard to the first, the negative answer is the justification of the operation of extirpation still so generally resorted to, and the motive for urging operative interference as early as possible. It implies a more or less confident belief in the local significance of the primary legion, and it is not inconsistent with the circumstance that, practically, the majority of tumors, which on extirpation prove to have the anatomy of cancer, do in fact recur, for the incomplete extirpation of marginal portions of the primary growth, or the existence already at the time of the operation of small secondary growths in distant organs, will sufficiently account for this result without any more violent supposition.

"On the other hand the negative answer to the second question might have been anticipated on purely anatomical grounds, since it would seem that in every cancer there must be a period when the small-celled infiltration of the connective tissue, and perhaps some increase in the number of the epithelial elements of any glandular part involved, is all that has taken place, and whenever this process commences simultaneously in a comparatively large area, instead of in a small one—in the whole mammary gland, for example, instead of in a small part of it—the size of a tumor may lead to its extirpation before its anatomy has become characteristic.

"As for the more ambitious efforts to combine together the anatomical facts and the clinical phenomena, so far as either are known, and to frame a comprehensive theory which shall embrace the whole, we must, for the present, regard them as somewhat premature; but it is a hopeful sign that even in such speculations the mythical notions of specific dyscrasiæ and heterologous new formations are being dropped more and more out of sight, and that we are learning more and more to endeavor to explain even the most aberrant phenomena of such a disease as cancer by the ordinary normal laws of development and growth."

